# Henipaviruses Employ a Multifaceted Approach to Evade the Antiviral Interferon Response

**DOI:** 10.3390/v1031190

**Published:** 2009-12-08

**Authors:** Megan L. Shaw

**Affiliations:** Department of Microbiology, Mount Sinai School of Medicine, New York, NY 10029, USA; E-Mail: megan.shaw@mssm.edu; Tel.: +1-212-241-8931; Fax: +1-212-534-1684

**Keywords:** Nipah virus (NiV), Hendra virus (HeV), zoonotic virus, interferon (IFN), STAT1, mda-5, nuclear localization

## Abstract

Hendra and Nipah virus, which constitute the genus *Henipavirus*, are zoonotic paramyxoviruses that have been associated with sporadic outbreaks of severe disease and mortality in humans since their emergence in the late 1990s. Similar to other paramyxoviruses, their ability to evade the host interferon (IFN) response is conferred by the P gene. The henipavirus P gene encodes four proteins; the P, V, W and C proteins, which have all been described to inhibit the antiviral response. Further studies have revealed that these proteins have overlapping but unique properties which enable the virus to block multiple signaling pathways in the IFN response. The best characterized of these is the JAK-STAT signaling pathway which is targeted by the P, V and W proteins via an interaction with the transcription factor STAT1. In addition the V and W proteins can both limit virus-induced induction of IFN but they appear to do this via distinct mechanisms that rely on unique sequences in their C-terminal domains. The ability to generate recombinant Nipah viruses now gives us the opportunity to determine the precise role for each of these proteins and address their contribution to pathogenicity. Additionally, the question of whether these multiple anti-IFN strategies are all active in the different mammalian hosts for henipaviruses, particularly the fruit bat reservoir, warrants further exploration.

## 1. Overview of the *Henipavirus* genus

The *Henipavirus* genus is part of the paramyxovirus family and consists of two members; Hendra virus (HeV) and Nipah virus (NiV) [1]. HeV was first described in 1994 and was associated with an outbreak of disease in horses on the East coast of Australia [2]. Humans in contact with infected horses were also at risk of infection and two people died during this 1994 outbreak, with another two deaths being reported during more recent outbreaks in 2008 and 2009. Nipah virus first emerged in 1998 in Malaysia where it was responsible for a large outbreak of disease in pigs as wells as humans that worked in the pig industry [3]. In humans, NiV infection causes acute encephalitis and 105 deaths out of 265 cases were recorded in this first outbreak. Since then there have been almost yearly reports of human infections with NiV from Bangladesh, with mortality rates around 70% and evidence of more respiratory disease [4]. HeV and NiV have both been isolated from bats belonging to the *Pteropus* genus (also known as flying foxes) and these animals are considered to be the natural reservoir for henipaviruses [5,6]. In Bangladesh there has been no evidence for an intermediate animal host, so direct bat-to-human transmission is suspected but there is also increasing evidence for human-to-human transmission [7]. In addition to bats, humans, horses and pigs, NiV infections of dogs and cats have also been reported indicating the wide host range for henipaviruses. This also makes them the first examples of zoonotic paramyxoviruses, which together with the high virulence in humans and lack of effective therapy, has resulted in them being classified as biosafety level 4 pathogens.

## 2. Henipavirus P gene products

### 2.1. mRNA editing

**Figure 1.**
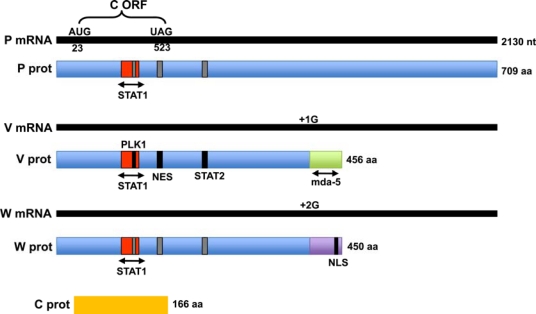
Illustration of the four protein products of the NiV P gene. The P, V and W mRNA transcripts are shown in black with the encoded proteins shown below. The C protein (encoded by the alternate ORF shown in the P transcript) is depicted in yellow. The unique C-termini in V and W, representing the +1 and +2 frames, are shown in green and purple, respectively. Functional domains that control protein localization or protein interactions are noted. The STAT1 binding domain within the N-terminal portion of P, V and W proteins is shown in red. Domains that have been characterized only in the context of the V protein are shown in black on the V protein and in gray for the corresponding positions on the P and W proteins. NES = nuclear export signal, NLS = nuclear localization signal.

Henipaviruses, as other members of the *Paramyxovirinae* subfamily, extend the coding capacity of their genome via an mRNA editing mechanism that gives rise to multiple proteins from the P gene. During transcription of the P gene the polymerase stutters at a run of A and G residues referred to as the editing site and this results in the addition of non-templated G residues into the nascent mRNA. An unedited henipavirus P mRNA encodes the phosphoprotein (P), which participates in viral RNA synthesis as a cofactor for the polymerase. The insertion of one extra G residue shifts the frame and this mRNA encodes the V protein (Figure 1). Insertion of two G residues accesses the +2 frame and this transcript codes for the W protein. NiV and HeV have been shown to edit their P genes at a particularly high frequency, with as many as 14 G insertions observed [8,9]. The overall ratio of P:V:W transcripts in NiV-infected cells is approximately 1:1:1 however it has been noted that at early times there is a higher proportion of P transcripts and as the infection progresses, the number of V and W transcripts exceed that of P [8]. The regulation of this process remains to be investigated and it is not yet known whether the same phenomenon can be observed with other paramyxovirus infections. Also, due to the high degree of editing, different forms of P, V and W are expected to be produced that contain additional glycine residues and it is not known if these lengthened forms have any altered function.

### 2.2. Domain structure and localization of the P, V and W proteins

As a result of the frame-shift that occurs after editing, the P, V and W proteins share the same N-terminal domain but have unique C-terminal domains (Figure 1). The common N-terminal domain is approximately 100–200 amino acids longer than that of its morbillivirus and rubulavirus counterparts, which is one of the distinguishing features of henipaviruses. The extreme N-terminus (up to residue 137) of the HeV and NiV P proteins is highly conserved and deletion mapping indicates that residues 81–120 in the NiV P protein are crucial for viral polymerase activity [10]. The remaining portion of the N-terminal domain shows only 43% identity between NiV and HeV [11]. This may go along with the finding that the N-terminal portions of paramyxovirus P proteins are natively unfolded and are likely to become structured only when induced to do so upon binding to an interaction partner [12]. This allows for incredible flexibility and one can speculate that the increased length of the henipavirus P (as well as V and W) proteins may be related to binding of cellular proteins that facilitate replication in multiple mammalian hosts.

The C-terminal domain of the HeV and NiV P protein is conserved and plays an essential role in replication as it mediates interaction with the polymerase and the N protein [13]. The C-terminal portion of the V proteins of henipaviruses and all other paramyxoviruses is very highly conserved and is characterized by the presence of seven cysteine residues. The C-terminus of the henipavirus W proteins is another feature unique to these viruses. In the morbilli- and respiroviruses the W ORF ends shortly after the editing site, essentially producing a protein representing the N-terminal domain of P. In contrast, the henipavirus W ORF extends for 43 amino acids which creates a unique C-terminus. Within the C-terminus of the NiV W protein (and conserved in HeV W) lies a basic stretch of amino acids that serves as a nuclear localization signal (NLS) (Figure 1) [14]. This has been shown to mediate specific interactions with karyopherins α3 and α4 [14], which are related members of the nuclear import machinery. The NiV W protein has been shown to be exclusively nuclear both in infected cells and when expressed from a plasmid in isolation of other viral proteins [9,14,15]. In contrast, the P and V proteins are both found in the cytoplasm [9,15–18]. In infected cells P co-localizes with the N protein, consistent with its essential role as part of the viral replication complex [9]. The common N-terminal domain of P, V and W has been shown to contain a nuclear export signal (NES - residues 174–192) and in the context of the V protein, deletion of this signal results in diffuse staining in both the nucleus and cytoplasm [17]. This would indicate that V can shuttle between these two compartments but is predominantly cytoplasmic. Presumably the NLS at the C-terminus of W overrides the export signal in its N-terminal domain, possibly due to a structural conformation that masks the NES. The V and W proteins have also been shown to inhibit NiV mini-genome activity which suggests a role in regulating viral RNA synthesis [19]. Along with the P protein, both V and W have been detected in preparations of purified NiV particles indicating that they can be packaged and therefore delivered to the new target cell upon infection [9].

There is limited information on post-translational modifications of P, V and W. As expected, the P protein is phosphorylated and specific sites have been identified in HeV P (Ser-224 and Thr-239) and NiV P (Ser-240 and Ser-472) [20]. It is not clear whether the sites located in the N-terminal region are also phosphorylated in the corresponding V and W proteins and if so how this may affect their function. The HeV and NiV V proteins are also described to be phosphorylated by polo-like kinase (PLK1), whose binding is dependent on prior phosphorylation of V at a particular site [21]. Interestingly, the HeV and NiV V have distinct PLK1 binding sites both of which lie in the N-terminal domain. In addition there is now evidence that PLK1 binding and phosphorylation of the parainfluenza virus 5 P protein is involved in regulating viral replication, so there is the potential for a similar role with henipaviruses, but this still has to be explored [22]. The other modification described for both HeV and NiV P proteins is N-terminal acetylation [20].

### 2.3. The C protein

The fourth protein encoded by the henipavirus P gene is the C protein, which is expressed from an alternate ORF present in P, V and W transcripts. This 18 kDa protein localizes to the cytoplasm in a punctate pattern and it can be detected at low levels in NiV virions [9]. Interestingly, a recombinant virus that lacks the C ORF shows attenuated growth properties in cell culture, which suggests that while not essential for replication, this protein does facilitate virus growth [10]. Possibly this attenuation is due to a regulatory defect, as the NiV C protein has been demonstrated to inhibit NiV minigenome replication [19]. Other paramyxovirus C proteins that have this property are those from measles virus, human parainfluenza virus 3 and Sendai virus.

## 3. Inhibition of interferon synthesis by henipavirus V and W proteins

**Figure 2.**
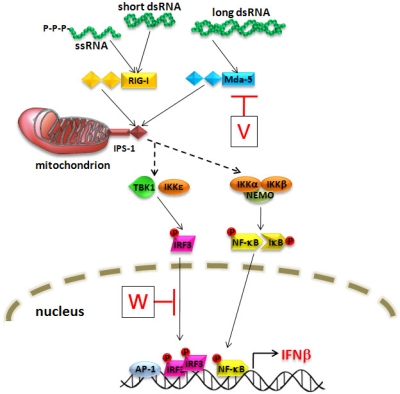
Illustration of virus-activation of IFN-β synthesis and its inhibition by the henipavirus V and W proteins. Detection of viral RNAs by RIG-I and mda-5 activates a signaling cascade though IPS-1 leading to the phosphorylation of IRF3 and NFκB. The activated transcription factors translocate to the nucleus and induce synthesis of IFN-β. The V protein prevents signaling by interacting with mda-5, while the W protein interferes with the activated form of IRF3 in the nucleus.

Cellular detection of virus infection is the trigger for initiating synthesis of IFN-β, and the molecules described to serve as cytoplasmic sensors for RNA viruses are RIG-I and mda-5 [23,24]. The natural ligands for these receptors are viral RNAs and they are characterized by a helicase domain and a caspase recruitment domain (CARD). This latter domain mediates an interaction with the mitochondrial-bound protein IPS-1 (also known as VISA/MAVS/Cardiff), which triggers a downstream signaling cascade to activate the transcription factors IRF3, NF-κB and AP-1 (Figure 2). Upon translocation to the nucleus these activated forms bind to the promoter and induce IFN-β synthesis. A similar induction of IFN-β occurs upon stimulation of some Toll-like receptor (TLR) members [24] and both TLR and RIG-I-like signaling pathways are targeted by viruses as a means to suppress activation of the host antiviral response [25].

### 3.1. Interaction of the V protein with RNA helicases, mda-5 and LGP2

Paramyxovirus V proteins limit the synthesis of type I IFN via a highly conserved mechanism [26]. A study using a recombinant PIV5 provided a clue that this function could be attributed to the C-terminal domain of V, as a virus lacking this domain induced greater levels of IFN-β than the wild-type PIV5 [27]. Reporter assays have shown that the block is at the level of transcriptional activation, as the V proteins of a number of paramyxoviruses, including NiV [14], can prevent activation of the IFN-β promoter in response to cytoplasmic dsRNA [26]. This is also true of other IRF3-dependent promoters (e.g., ISG54, ISG56), indicating that the signaling pathway leading to IRF3-activation is being targeted [14]. For NiV, it has been shown that V is unable to prevent IRF3 activation in response to TLR3-mediated signaling, which suggested that it is acting specifically on the virus- or intracellular dsRNA-trigged pathway for IFN induction. The first description of the RNA helicase, mda-5, and its signaling role in the IFN induction pathway provided an explanation for these early findings [28]. It was found that the V proteins can interact with mda-5 via their cysteine-rich C-terminal domains and thereby prevent downstream signaling and hence, activation of the IFN-β promoter (Figure 2) [28,29]. V acts by binding to the helicase domain of mda-5 through a minimal region encompassing residues 701–816 [30,31]. In one study this interaction was shown to prevent binding of dsRNA and subsequent oligomerization of mda-5 [30], while another indicated that ATP hydrolysis is inhibited [31]. The paramyxovirus V proteins can also bind to an analogous domain in the helicase portion of LGP2 [31]. LGP2 lacks the N-terminal CARD domain and therefore is not an active signaling molecule but is thought to play a role in the regulation of IFN induction by RIG-I and mda-5 [23]. The role of the V:LGP2 interaction in the IFN response remains to be determined.

The V proteins are notably unable to interact with or inhibit signaling from the related RNA helicase, RIG-I [29]. Due to the more pronounced effect of RIG-I deficiency on virus induction of IFN, RIG-I is thought to be the predominant sensor for most viral infections. Thus it is unclear whether the lack of V-mediated inhibition implies that RIG-I is not involved in sensing paramyxovirus infections or whether paramyxoviruses can target RIG-I via additional proteins. That said, there are reports of mda-5 being important for the detection of Sendai virus defective interfering particles in dendritic cells [32], so assessing the true biological role of mda-5-inhibition may be a limitation of the tools at hand.

### 3.2. W-mediated inhibition of IFN production from the nucleus

Like the V protein, the W protein of NiV can also prevent activation of the IFN-β promoter that is triggered by virus or intracellular dsRNA [14]. As W has a completely different C-terminal domain from V, it does not contain the mda-5 interaction motif, so it must act via an alternative mechanism. NiV W also has the ability to interfere with TLR3 signaling, which V is unable to do [14]. These data indicate that the W protein can target IFN synthesis via a unique mechanism. The other unique feature of W is its nuclear localization and it was shown that a mutant W protein that lacks its NLS loses the ability to block TLR3 signaling [14]. Correspondingly, if the V protein is artificially targeted to the nucleus, it gains the ability to inhibit TLR3-mediated signaling. These data show firstly, that nuclear localization is critical for this activity and secondly, that the domain responsible for mediating the inhibition must lie within the common N-terminal domain shared by V and W. The TLR3 and virus-activated signaling cascades that lead to induction of IFN-β involve unique signaling molecules but at some point they converge and activate the same set of transcription factors required for activating IFN-β gene expression. Therefore if W is acting in the nucleus it is conceivable that it is interfering with a final step in the pathway that is required for signaling events derived from multiple sensors. IRF3 is known to be a critical component and the presence of phosphorylated forms in the nucleus is used as a signature for an active IFN response. In the presence of NiV W IRF3 is phosphoylated in response to TLR3 signaling, however as the amount of W is increased there is an accompanying loss of the activated form of IRF3, which is not seen with the V protein [14]. This implies that W does not block activation of IRF3 but that once this active form is in the nucleus it is less stable in the presence of NiV W which prevents proper activation of the IFN-β promoter (Figure 2). Clearly, the precise mechanism by which NiW acts remains to be determined but it would appear that the presence of an NLS in its unique C-terminus, and hence an altered distribution compared to V, confers on this protein an additional means by which to inhibit the IFN response. As NiV and HeV are distinct in having W proteins with extended C-termini, this may indicate that this additional anti-IFN mechanism is unique to the henipavirus genus.

## 4. Inhibition of interferon signaling by henipavirus P, V and W proteins

**Figure 3.**
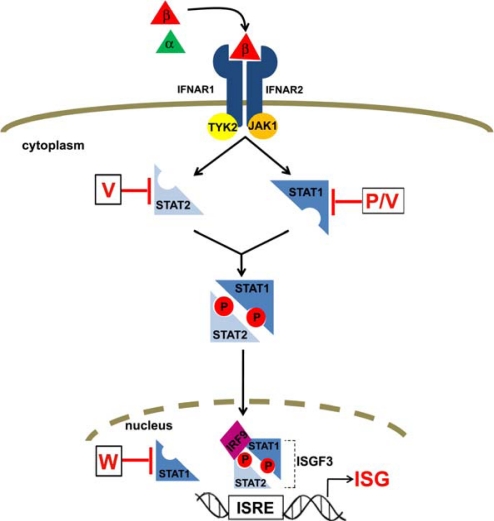
Illustration of the type I IFN signaling pathway and its inhibition by the henipavirus P, V and W proteins. IFN-α/β binds to the IFN-α/β receptor (composed of IFNAR1 and IFNAR2 subunits). The receptor-ligand interaction activates the Janus protein tyrosine kinases, TYK2 and JAK1, which in turn activate the STAT1 and STAT2 transcription factors via tyrosine phosphorylation. The phosphorylated STATs form a heterodimer and translocate to the nucleus where together with IRF9, they form the ISGF3 (IFN stimulated gene factor 3) complex. This transcription factor complex binds to IFN-stimulated response elements (ISRE) and activates transcription of IFN-stimulated genes (ISG). The P and V proteins bind to STAT1 (and STAT2 for V) in the cytoplasm and prevent its activation in response to IFN. The W protein acts via the same mechanism but it sequesters STAT1 in the nucleus in an inactive form.

Both type I (α/β) and type II (γ) interferons (IFN) mediate their effects on cells by binding to receptors on the cell surface and activating the JAK-STAT signaling pathway [33]. STAT (Signal Transducers and Activators of Transcription) proteins are transcription factors that are mainly cytoplasmic in their inactive state. In response to IFN-α/β, STAT1 and STAT2 become tyrosine phosphorylated, dimerize, and translocate to the nucleus (Figure 3). The activated heterodimer complexes with interferon regulatory factor (IRF) 9 to form the ISGF3 (IFN stimulated gene factor 3) complex. ISGF3 binds to IFN-stimulated response elements (ISRE) located in the promoters of IFN-stimulated genes (ISG) and activates transcription leading to the production of many proteins that have antiviral activity. For this reason many viruses have mechanisms to inhibit the JAK-STAT signaling pathway and paramyxoviruses in particular have a preference for targeting the STAT proteins [34]. Interestingly, they do so via distinct mechanisms and the henipaviruses act by sequestering STATs and preventing their activation in response to IFN.

### 4.1. STAT re-localization and inhibition of IFN-activated phosphorylation

In the presence of either NiV P, V or W proteins, there are reduced levels of tyrosine 701 phosphorylated STAT1 in IFN-α/β-treated cells [15,18]. This lack of STAT1 phosphorylation corresponds with an inhibition of ISRE-driven gene expression in cells that are expressing these proteins, indicating that the IFN signaling pathway is not being activated [15,18,35]. STAT1 is also required for IFN-γ-mediated signaling and this pathway is also blocked by expression of both HeV and NiV V proteins [16,18], and presumably also the P and W proteins. The finding that P, V and W share this activity suggests that the domain responsible lies within the common N-terminal region and indeed, when this domain is expressed alone it too shows the ability to block IFN signaling [15,35]. On an individual basis though, it has been shown that at limiting concentrations, P has the weakest activity and W the strongest, suggesting that the properties of the full-length proteins can modulate this activity [15].

In an unstimulated cell STAT1 shuttles between the cytoplasm and nucleus but upon IFN treatment, the phosphorylated and dimerized form translocates to the nucleus. In cells that are expressing P and V proteins, STAT1 is retained exclusively in the cytoplasm in its inactive, non-phosphorylated form even in the presence of IFN-α/β or IFN-γ [15,16,18]. The V proteins of both NiV and HeV have also been shown to sequester STAT2 in the cytoplasm and prevent its nuclear accumulation in response to IFN-α/β [16,18]. However, in cells expressing the W protein, the inactive form of STAT1 is re-localized to the nucleus [15]. The status of STAT1 in NiV-infected cells has recently been examined and there is no evidence of phosphorylated STAT1, which correlates with the plasmid-expression data and indicates that IFN signaling is not active in infected cells [10]. Interestingly the inactive form of STAT1 is completely localized to the nucleus in the infected cells but remains cytoplasmic in non-infected cells [10]. This mimics the pattern seen in W-expressing cells and suggests that the NiV W protein is most likely mediating this effect in infected cells.

### 4.2. Interaction with STAT1 and STAT2

As suggested by the strong co-localization data, the P, V and W proteins can all interact with STAT1 [10,15–18]. The V protein has also been shown to interact with STAT2 and in the presence of V, both STAT1 and STAT2 are found in high-molecular weight complexes, which indicates that they are sequestered into a large, multi-protein complex [16,18].

**Figure 4.**
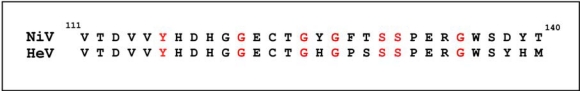
An alignment of the STAT1 binding domain (residues 111–140) in the P, V and W proteins of NiV and HeV. Those residues that have been shown to be critical for the STAT1 interaction are shown in red.

The STAT1 binding domain lies within the N-terminal portion of P, V and W [10,15,17]. Finer mapping has shown that a 30 amino acid region spanning residues 111–140 of P/V/W is critical for the interaction (Figure 4) and that loss of binding correlates with an inability to prevent STAT1 phosphorylation and to inhibit IFN signaling [10]. A glycine at position 125 within this binding domain has been implicated in IFN signaling inhibition as the V protein from a human isolate of NiV which contains a glutamic acid at this position was shown not to bind to STAT1 or STAT2 [36]. Ciancanelli *et al.* [10] examined the contribution of surrounding glycine residues within the binding domain and in addition to confirming the importance of G125, they reported that substitution of glutamic acid for glycines at positions 121, 127 and 135 (but not 120) could all abrogate STAT1 binding in the context of P, V and W proteins. This correlated with the ability to prevent IFN signaling, although the G135E mutant retained more activity than the other mutants despite not visibly interacting with STAT1. The contribution of tyrosine 116 within the defined STAT1 binding domain was also examined due to the known requirement of a tyrosine within a similar motif in the measles virus P protein for STAT1 inhibition [37]. Mutation of tyrosine 116 in NiV P to either alanine or glutamic acid resulted in loss of IFN inhibitory activity and loss of STAT1 binding [10]. Interestingly, a P protein with a Y116F substitution retains the ability to block IFN signaling and to bind STAT1, indicating the requirement for an aromatic residue at this position. Another study addressed the contribution of residues that make up an SSP motif in the N-terminal domain of P, V and W. These residues lie within the STAT1-binding domain and, in the context of the NiV V protein, have been shown to mediate an interaction with PLK1 [21]. For both NiV and HeV V proteins, alanine substitution of the serine residues at positions 130 and 131 resulted in loss of STAT1 and STAT2 binding activity and a corresponding loss of ability to inhibit both IFN-α/β and IFN-γ signaling. For position 130, substitution with threonine did not disrupt STAT binding despite the fact that this mutation does eliminate the interaction of NiV V with PLK1. These and other supporting data led to the conclusion that V-mediated interactions with STAT proteins and PLK1 are separable functions [21].

In order to verify the importance of the residues conferring STAT1-binding within the context of the whole virus, it was necessary to determine whether they were also required for the polymerase co-factor function of the P protein. Using a NiV mini-genome assay to assess polymerase function, it was shown that the critical region for RNA synthesis lies within amino acids 81–113 of the P protein and that substitutions that affect STAT1 binding do not interfere with polymerase activity [10,21]. This information allowed the construction of a recombinant NiV that lacked STAT1 binding activity due to a G121E mutation in the P, V and W proteins [10]. As this region overlaps with the C ORF and would result in an amino acid change in C, this virus was engineered in a C knockout background. NiV C^KO^ and C^KO^/G121E viruses display identical growth properties in both 293T and Vero cells, although interestingly both are attenuated relative to the wild-type NiV. Crucially, infection with the C^KO^/G121E virus induces STAT1 phosphorylation in response to IFN-β whereas WT and C^KO^ viruses do not [10]. Also, normal nuclear translocation patterns for STAT1 are observed in NiV C^KO^/G121E infected cells in response to IFN. In contrast the inactive form of STAT1 remains sequestered in the nuclei of NiV WT and C^KO^ infected cells. As mentioned earlier, these data suggest that the nuclear W protein is functioning as the main obstacle to IFN signaling and that this is abolished by the G121E mutation, which eliminates the STAT1 interaction. The finding that the recombinant NiV lacking STAT1-binding has identical growth properties to its C^KO^ parent virus, even in 293T cells (which have a functional IFN response), indicates that the lack of STAT1 inhibition is not detrimental to the virus. This suggests that additional anti-IFN mechanisms encoded by NiV are probably still intact. These could be either V-mediated inhibition of mda-5, W-mediated inhibition of IRF3 activity in the nucleus, or both acting in concert.

To date, only the V protein has been reported to interact with STAT2 [16,18,36]. This interaction is dependent on STAT1 as it has been shown that in STAT1 negative cells, there is no V:STAT2 interaction and so far all mutations in V that eliminate STAT1 binding have resulted in a simultaneous loss of STAT2 binding [17,21,36]. However, Rodriguez *et al*. [17] also demonstrated that a NiV V protein lacking residues 230–237 was deficient in STAT2-binding despite being able to interact with STAT1. This led them to the conclusion that for STAT2 to interact with V, both the STAT1 binding site and this STAT2-specific site are required. The precise role for the STAT2 interaction remains unclear, as the ability to prevent IFN signaling appears to be strictly dependent on STAT1 binding and inhibition.

Currently, there is limited information regarding the region of STAT1 that interacts with the NiV proteins. The domain structure of STAT1 can be broadly divided into an N-terminal domain, a coiled-coiled region, a DNA-binding domain, a linker domain, an SH2 domain and a transactivation domain [38]. Using a set of STAT1 and STAT3 (which does not interact with V) chimeric constructs, it has been possible to map the NiV V-interaction site to amino acids 509–712 on STAT1 [17]. This incorporates the linker domain and the SH2 domain as well as tyrosine 701 which is the residue phosphorylated in response to IFN.

## 5. The unexplored role of the C protein

Of all the henipavirus P gene products, we know the least about the C protein. Plasmid-based expression of the NiV C protein has been shown to prevent the induction of a robust antiviral response [35] but the mechanism by which it acts is unknown. Perhaps the strongest indication for the importance of the C protein is shown by the recombinant NiV C^KO^ virus which has attenuated growth properties relative to the wild-type virus [10]. The fact that this is seen in Vero cells as well suggests that this phenotype is not mediated by IFN and it may reflect the role of the C protein in regulating viral RNA synthesis [19].

## 6. Conclusions

In conclusion, through their unique coding strategy, henipaviruses produce multiple proteins that antagonize the antiviral response at multiple levels. While the shared N-terminal domains of the P, V and W proteins direct inhibition of STAT signaling, the C-terminal domains of the V and W proteins confer unique properties on these proteins that extend their antagonist function. For V this involves mda-5-binding and for W this involves nuclear localization and the ability to block a late stage of the IFN induction pathway. The question of whether all these mechanisms are active in an infected cell is something that still needs to be explored. Unfortunately the need for biosafety level 4 conditions has limited this work but the description of a reverse genetics system for NiV opens the door to addressing the contribution of each protein and/or mechanism. Another factor to consider is that the presence of multiple anti-IFN mechanisms may relate to the zoonotic nature of these viruses. Even though it has already been shown that the NiV V protein can prevent IFN signaling in cells from multiple species [36], some of the other viral proteins may have species-specific activity. It is of particular interest to explore this possibility in pteropid bats, which are the natural reservoir of henipaviruses and from all accounts seem to control virus infection far better than other mammalian hosts [39].
